# Diverging from News Media: An Exploratory Study on the Changing Dynamics between Media and Public Attention on Cancer in China from 2011–2020

**DOI:** 10.3390/ijerph18168577

**Published:** 2021-08-13

**Authors:** Yangkun Huang, Xiaoping Xu, Sini Su

**Affiliations:** 1 School of Media and Communication, Shanghai Jiao Tong University, Shanghai 200240, China; koan1997@sjtu.edu.cn; 2 School of Journalism and Communication, Lanzhou University, Lanzhou 730000, China; xuxp@lzu.edu.cn; 3 College of Media and International Culture, Zhejiang University, Hangzhou 310058, China

**Keywords:** cancer, public attention, news media, Granger causality test, data mining

## Abstract

Over the past decade, China has witnessed fast-paced technological advancements in the media industry, as well as major shifts in the health agenda portrayed in the media. Therefore, a key starting point when discussing health communication lies in whether media attention and public attention towards health issues are structurally aligned, and to what extent the news media guides public attention. Based on data mined from 73,060 sets of the Baidu Search Index and Media Index on 20 terms covering different types of cancer from 2011 to 2020, the Granger test demonstrates that, in the last decade, public attention and media attention towards cancer in China has gone through two distinct phases. During the first phase, 2011–2015, Chinese news media still held the key in transferring the salience of issues on most cancer types to the public. In the second phase, from 2016–2020, public attention towards cancer has gradually diverged from media coverage, mirroring the imbalance and mismatch between the demand of active public and the supply of cancer information from news media. This study provides an overview of the dynamic transition on cancer issues in China over a ten-year span, along with descriptive results on public and media attention towards specific cancer types.

## 1. Introduction

Cancer universally ranks as a leading cause of death and is a crucial barrier to increasing life expectancy [1]. In 2020, the estimated number of new cancer cases was 19.29 million globally, and approximately one out of every four new cancer patients appear in China [2]. This life-threatening and increasingly prevalent disease has been a highly explored topic when it comes to the cross-field of cancer and health-information seeking behavior (HISB) research [3,4,5,6].

HISB is widely defined as all the ways in which individuals go about obtaining health information [7], from which some sub-concepts have been derived, e.g., internet health-information seeking behavior (IHISB) and online health-information seeking behavior (OHISB). Generally, self-reported questionnaires or interviews, and data mined from search engines and other websites are the main two data sources used in HISB studies. Web data mining is now increasingly adopted over self-report methods, because of its high efficiency, comprehensiveness, and objectivity, which address the major drawbacks of the latter [8].

It should be noted that there have been arguments on what constitutes a sign of public attention. Data from surveys with traditional deployment methods were once accepted means to assess public attention and opinions, however over the years it has become evident that such data fails to capture the elusive dynamics of public attention [9]. On the other hand, Eysenbach posits that log data from search engines “allow valuable insights into information needs and human behavior” and “can be meaningful inferences made on the presumed intention of the user ” [10]. Experts in other fields have also provided support for this idea with empirical studies [11,12]. As Web-service-generated data represent a gradually increasing non-negligible instantiation of public attention, more and more scholars in health, policy and politics, media and communication, as well as other disciplines across the social and natural sciences, are using online search data to quantify public attention [13,14,15].

In recent years, the cancer-related agenda has changed within Chinese media. According to the reversed agenda-setting hypothesis [16,17], the public is no longer a passive party but are in fact so-called active users, independent of media agenda-setting [9]. So it follows to ask whether the news media can still lead public attention as it did in the era of mass communication [18,19]. Public attention on cancer not only promotes health consciousness and reduces people’s risk of disease, but has also been proven to be a significant predictor of social support [20,21], with great relevance to personal health and social harmony in the long run. Thus far, however, there has been little research on this topic in China, a country with the world’s largest cancer population. Baidu, owing to its position as the largest Chinese search engine globally, offers an effective platform for exploring cancer-information seeking behavior (CISB) in China.

Based on data mined on the Baidu Search Index and Media Index of 20 cancer types from 2011 to 2020, the study articulates the distribution of public and media attention towards different cancer categories, and clarifies the lead-lag patterns, namely the causal linkage, between media reports and public seeking behavior for cancer information in China. The remainder of this article is structured as follows: Section 2 explains the data source, data acquisition method, and the overarching research design; Section 3 presents statistical outcomes, including descriptive statistics and Granger causality results; The conclusion and discussion that are central in the research, have been given separately in Section 4 and Section 5.

## 2. Materials and Methods

**Figure 1:**
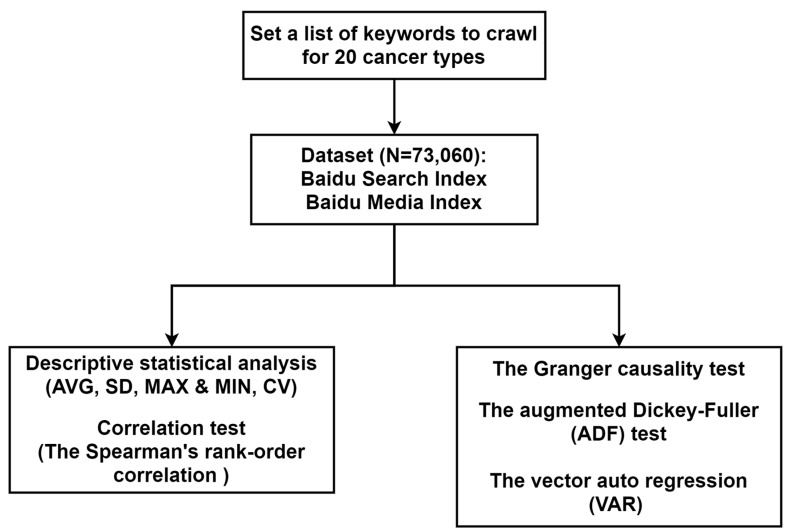
The flowchart of data obtaining and analysis.

This study employed a set of quantitative methods (Figure 1). Descriptive statistics and correlation tests were performed to determine how Chinese media, as well as Chinese netizens, have allocated attention in terms of specific cancers in the last decade, reflected by search engine data and China’s latest cancer registry data. Then the Granger causality test was adopted apropos of the causal inference between time series [22]. To avoid spurious regression, the augmented Dickey-Fuller (ADF) test was used first, and non-stationary variables were processed by taking the difference so as to ensure stationary data [23]. Furthermore, the vector auto regression (VAR) was combined to determine the lag specification according to the information criterion [24]. EViews 11.0 was used for the Granger causality test alongside the ADF test and VAR modeling, coupled with SPSS 25.0 for the correlation coefficient and the coefficient of variance (CV) calculations.

The data used in the analysis were the Baidu Search Index and the Media index from Baidu, a Chinese tech company analogous to Google. From 2011 to 2020, Baidu accounted for the bulk of the domestic search engine market share and has been empirically proven to be the most commonly-used tool for seeking cancer-related information among Chinese netizens [25,26,27]. Furthermore, the search engine embraces a kaleidoscope of electronic news content from both state-owned and pro-business Chinese media, providing a window into the dynamic flow of media attention towards cancer.

To be specific, the Baidu Media Index is based on the quantity of news reports from major internet media, related to keywords and concurrently collected by Baidu News Channel, whereas the Baidu Search Index takes keywords as statistical objects and calculates the public search volume for a certain keyword.

Due to the fact that cancer is not a single disease but a multiplicity of variously related diseases, 20 cancers were considered as research targets to cover as many types of cancer as possible, including: (1) lung cancer, (2) head and neck cancer, (3) oesophageal cancer, (4) stomach cancer, (5) colorectal cancer, (6) liver cancer, (7) pancreatic cancer, (8) skin cancer, (9) breast cancer, (10) cervical cancer, (11) endometrial cancer, (12) ovarian cancer, (13) prostate cancer, (14) testicular cancer, (15) bladder cancer, (16) kidney cancer, (17) brain cancer, (18) thyroid cancer, (19) non-Hodgkin lymphoma, and (20) leukemia, all of which are cancer types or subtypes mentioned in the IARC (International Agency for Research on Cancer, IARC) World Cancer Report 2020 [28].

This study crawled the Baidu Search Index and Media Index of the 20 cancers terms listed above from 2011 to 2020 using Python code. This process confirmed that all the cancers listed had corresponding index data in Baidu. In total, 73,060 sets of index data were collected and the date of data collection was 5 January 2021.

It is important to note that, considering the potential effect of fraudulent traffic generated by web bots and the filter bubble, we have done a certain amount of work. Firstly, we collected and checked Baidu’s statements on the bot traffic and their Robots Exclusion Protocol, confirming that there are systemic anti-crawler strategies in Baidu, and especially, that Baidu has formulated strict rules against those cheating methods of data fraud, managing to maintain the fairness and impartiality of all indices data. Secondly, to examine the existence of cancer-specific filter bubbles, we designed a pre-test involving Baidu’s users from several places in China. This additional investigation revealed that, no matter whether participants have previous searching experience and pre-existing digital traces or not, there was no obvious difference both in their search results and news feeds on keywords for specified cancer types.

## 3. Results

**Figure 2:**
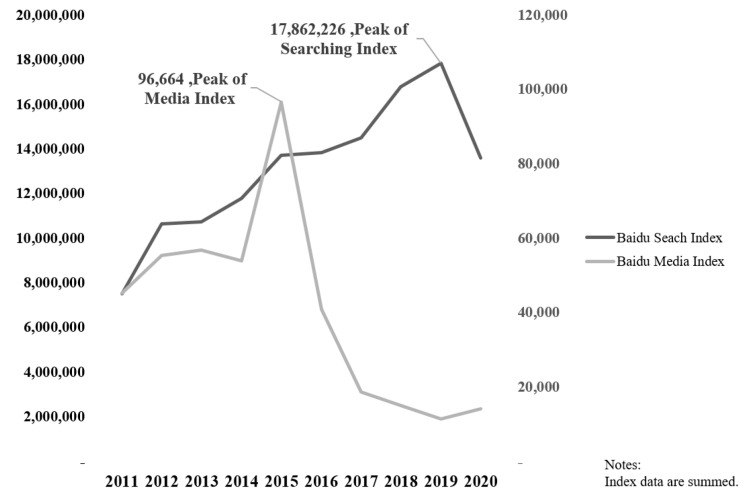
Yearly distribution of the two indices over ten years.

The distribution of the allocation of the public and media attention towards 20 cancer types by years has been mapped out in Figure 2. As is shown, the patterns of the two lines are completely different. The Baidu Search Index of the 20 kinds of cancer has maintained a long-term rising trend but in 2020, the year of the COVID-19 pandemic, declined to levels similar to that of the year 2015, while the Baidu Media Index has been hovering at a low level since a precipitous drop in 2015.

As Table 1 displays, the average Baidu Search Index of leukemia was the highest among the 20 cancers (M = 4734.769), and conversely, the lowest was head and neck cancer (M = 31.224). According to the latest data, leukemia was not even the top 10 most common cancers in terms of crude incidence rates and mortality rates [29,30], but the one most concerned by Chinese netizens.

In the past decade, the public’s searching activities have been relatively steady on the account that CV for CISB is less than 1, except for head and neck cancer and breast cancer. Breast cancer, with the highest maximum value 313,389, has undergone the most drastic change in public attention during the last decade. The Baidu Search Index for breast cancer reached its peak on 16 January 2015, the day that Bella Yao, a famous Chinese female singer, died of breast cancer. The incident itself, along with a succession of debates on media ethics, has gone down in the history of Chinese health communication [31,32,33].

According to the Spearman correlation test, the value of the Spearman’s Rho between the Baidu Media Index and the number of cancer cases (ρ = 0.556, *p* = 0.013 < 0.05) is higher than that of between the Baidu Media Index and the number of cancer deaths (ρ = 0.472, *p* = 0.041 < 0.05).

Briefly looking at the means of the Baidu Media Index (Table 2), we can conclude that the media attention on different cancer types was thoroughly out of balance between 2011–2020. Leukemia attracted extremely disproportionate media attention (M = 48.771), while head and neck cancer was still the least exposed subject of reports (M = 0.001), which perfectly corresponds to the situation of the Baidu Search Index.

As for whether it was the more fatal cancer or prevalent cancer that drew more public attention, the findings were similar to that of the Baidu Media Index, where the relationship between the Baidu Search Index and the number of cancer cases is much stronger (ρ = 0.472, *p* = 0.041 < 0.05). Whereas there is no statistically significant correlation between the Baidu Search Index and the number of cancer deaths (*p* = 0.198 > 0.05).

The Granger causality tests (Table 3) indicate causation between the Baidu Search Index and Media Index of the 20 cancers. Furthermore, the causal relationship of certain cancer types, both one-way and two-way, have been tested and found to occur at least once a year on a decennial scale.

Of the 20 cancers searched, stomach cancer was the only type for which the causal linkage between the two indices had never been broken. The Granger test showed that the Baidu Media Index of stomach cancer caused its Baidu Search Index for the most part, with the exceptions in 2013, 2017, 2019 (mutual causation) and 2020 (one-way Granger-causality from the Baidu Search Index to the Baidu Media Index). The second coherent link exists between the indices of Leukemia, which only discontinued in 2020.

**Figure 3:**
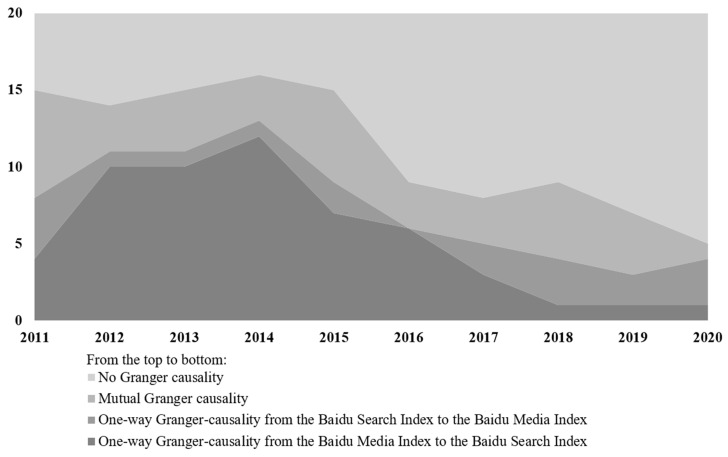
Frequency of occurrence of different Granger-causality relations.

It is demonstrated in Figure 3 that there were two phases, marked by the year 2015. The first phase was supportive of a media-generated agenda, and the second phase exhibiting divergent attention. The media had once, from 2012 to 2015 in particular, dominated the agenda-setting in China, deeply influencing the public agenda on cancer topics, and meanwhile, public attention also produced a sustained but very weak effect on what was discussed on the media newsrooms. As cancer news moved into the second half of the decade, especially in 2020, the agenda-setting ability of the media seems to have gradually taken a downturn. The causal linkage (one-way Granger-causality from the Baidu Media Index to the Baidu Search Index) was no longer significantly obvious, and mutually independent connections, or divergence between the Baidu Search Index and Baidu Media Index, emerged.

**Figure 4:**
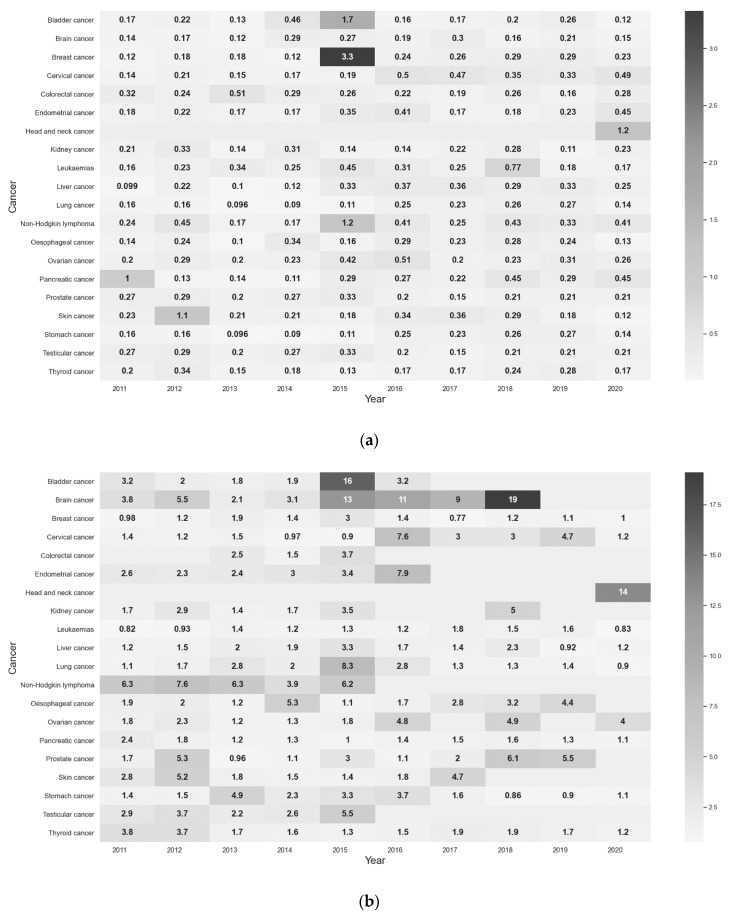
(**a**) Description of yearly CV of the Baidu Search Index among 20 cancers; (**b**) Description of yearly CV of the Baidu Media Index among 20 cancers.

To address the shortcomings of the Granger causality test, a supplemental CV calculation was performed to locate the strong variation in public attention, and then to determine if the cancer-related media events aroused public interest on the basis of the extent of variability in the Baidu Media Index. By calculating the CV by year, it is found in Figure 4 that in the past decade, compared with the Baidu Media Index’s dramatic shift, public attention to cancer has not fluctuated much, and the high variation (CV > 1) of the Baidu Search Index occurred six times; including pancreatic cancer in 2011, skin cancer in 2012, non-Hodgkin lymphoma, bladder cancer, and breast cancer in 2015, as well as head and neck cancer in 2020.

Among them, four fluctuations of the Baidu Search Index were related to the cancer diagnosis and death of celebrities (see Table 4), three of which reached their peak in the Baidu Media Index a few days before or just in sync with peaks in the Baidu Search index, perfectly predicting the steep increase in searching behavior. The same is also true in cases of cancer-related film screenings. However, such conditions have become rare since 2015, coinciding with the preceding result that 2015 marks the transition in the trend of the attentiveness from the public and news media.

## 4. Discussion

The news industry was previously regarded as the first port of call for valuable information and stories. Nowadays, with the aid of increasingly advanced technology, we all are ushering in a new era where the entry barrier to news reporting has been lowered, and anyone can be active in the generating and spreading of media content.

The field of health communication is no exception: Revolutionary strides in communication have profoundly influenced public health perception and behavior. News media, once thought of as the most trusted source of health information for the public in the past, is now confronted with numerous new challenges regarding access to health information. Hence, it is a concerning question, for both government departments and health organizations, whether news media can still perform the key role in reaching out to the public and leading their awareness on the health agenda—because if the answer is no, transformation and innovation in health advocacy and promotion should be formulated and adopted accordingly.

Our research begins with how the Chinese media and public have allocated their attention towards different types of cancer in the last ten years, and one of our primary findings is a descriptive overview of the current status. In the distribution of attention, both the media and the public tended to be concerned with cancers of higher incidence. The reason why the media exhibited such a preference is fully understandable as an effective media strategy, which should, after all, cover more people and strive for impact on a larger scale. However, the correlation coefficient of the Baidu Search Index did not reflect the relationship between the CISB and cancer-related death toll. This seemingly goes against our common sense and previous assumption, as the fear of cancer, which has a strong possibility of leading to the CISB [34], emanates from a core fear of mortality of such a sickness unto death [35]. With regard to the different cancer types, leukemia, with unimpressive morbidity and mortality, has been the top focus of the Chinese public and media over the previous decade, with the least attention directed to head and neck cancer, a general term for a variety of cancers.

What sets media attention apart is its much higher variation, where the CV of the Baidu Media Index of ten years and inter years mostly exceeds 1. Though the disparity in coverage may be reasonable in a practical sense, inasmuch as the time, resources, and manpower of news organizations are limited, such practice may have negative effects on health behavior and decision-making, considering the fact that an estimated millions of people suffer from these types of cancers, such as oesophageal and thyroid cancer, the incidence rates of which respectively ranked sixth and eighth in China [29,30].

Although previous studies revealed a marginally significant positive relationship between public attentiveness and journalistic pieces about cancer [18], few studies concentrate on the causal linkage in between. With Granger causality tests, ample evidence from the search engine addresses the question.

With the Granger tests, our findings more or less revisited the viewpoint of Russell et al. that there also exists “an interaction and differentiated resonance” among cancer attention in the first five years of the last decade [9]. The indices also indicate a diverging gap between the attention of the public and media, specifically in the year 2015, dividing the cancer-related communication into two phases. In the first phase, 2011 to 2015, the media’s strong agenda-setting function was highlighted by the fact that the Baidu Media Index of at least half of 20 cancer types Granger-caused the Baidu Search index, consistent with traditional agenda-setting theory which posits that the truncated versions of the world presented by the news media are a primary source of people’s perceptions of public affairs [36]. However, in the second phase, the media’s agenda-setting capabilities did not function nearly as well when their agenda was of little relevance to the concerns of the public, neither addressing nor catering to the demand for cancer information among the population over the last five years. This was especially the case in 2020 when the gap widening was accelerating, and the causal linkages between the attention from both the public and media were substantially fractured, as cancer information-seeking was quite likely heightened when under the conditions of an epidemic [37,38].

With recent technological advances, and the emergence of new media empowering users, it is no surprise that the news media’s agenda-setting ability has become a thing of the past and is no longer a leading indicator of public awareness regarding topics like cancer. But there was an unexpected yet thought-provoking result: reversed agenda-setting has not become mainstream and is still unconventional as Maxwell McCombs’s inference goes [39].

However, we cannot totally negate the agenda-setting function of news media on cancer, as the yearly CV calculation reflected the ability of the media to set the agenda on certain types of cancer-related issues. For example, the apparent fluctuations in information-seeking behavior were regularly caused by celebrity news and media events, i.e., (1) a Chinese Hong Kong actor Nicholas Tse’s diagnosis of skin cancer in 2012, (2) a Chinese singer Bella Yao’s death of breast cancer in 2015, (3) the screening of a film work *Go Away Mr. Tumour* with a storyline about Non-Hodgkin Lymphoma in 2015, and (4) a Chinese politician Xu’s death of bladder cancer in 2015, all of which were in agreement with previous findings that celebrity health disclosures and events can encourage the HISB [40,41]. Looking into a variety of news media consumed, the public showed more solicitude for the cancer coverage in entertainment and social news versus cancer in health and science reports.

Our study had some limitations. Firstly, though it revealed a divergence between the public attention and the news media agenda, there was no evidence to determine whether the public attention flowed to social media or somewhere else, which requires further discussion and research. Secondly, the cancer registry data of the China National Cancer Center was the most up-to-date cross-sectional data, but not a dynamic time series data based on the past decade. Thirdly, although we did not observe that Baidu’s cancer information feeds vary from person to person in the supplementary investigation, Baidu did develop the function of personalized recommendation, which can trap cancer information harvesters with probabilities in filter bubbles created by the search engines. The potential effects of filter bubbles can also strengthen or weaken the seekers’ activities for specific cancer information, leaving our conclusion questionable and future studies intriguing. Finally, Granger causality tests reflect the causation statistically, rather than in the philosophical sense, and additional research is needed for the exploration of causal linkage.

## 5. Conclusions

There was a much more distinct divergence between the public attention and media attention toward the cancer-related agenda in China, reflecting a mismatch between public cancer-related information demand and news media content generation, an imperative issue to tackle for effective public health communication. Causes of the divergence remain to be further discussed. Generally, we are inclined to regard it as a manifestation of the new media empowering users—users generate, disseminate, and consume health information without relying on the news agency of centralization. Then inconsistencies between public attention and media attention have been spontaneously pervading the information ecology. Yet we also cannot, solely through this study, single out the role of the elusive filter bubble of platforms and the collapsing public trust in news media, both of which, after all, can entail the fixation and shift of the public eye. We, thus, invite more in-depth thinking on attributions to this divergence.

But one thing that is certain is that there has been an existing divergence between the public and media in the past decade. There are substantial grounds to believe that COVID-19 is disrupting, and will continue to disrupt, cancer information acquisition where urgent access to medical recommendations has switched to telehealth [42]. Considering the public’s active demand for various types of cancer information and the Chinese news media’s less prominent position in setting the cancer agenda, media with the function of personalized agenda-setting, for instance, we-media and algorithmic media, should be given greater importance in the context of public health communication for cancer as a halting post-pandemic transition has already become the foregone conclusion.

## Figures and Tables

**Table 1:** Descriptive information of 20 cancers’ Baidu Search Index.

Cancer	Means	Standard Deviations	Maximum	Minimum	CV
Bladder cancer	1157.554	806.099	44,617	306	0.696
Brain cancer	635.727	189.219	3379	230	0.298
Breast cancer	3535.311	6357.250	313,389	951	1.798
Cervical cancer	4150.887	2820.908	24,280	491	0.680
Colorectal cancer	215.074	81.390	1544	0	0.378
Endometrial cancer	1019.366	369.607	4896	237	0.363
Head and neck cancer	31.224	154.585	3355	0	4.951
Kidney cancer	755.150	248.204	3169	206	0.329
Leukemia	4734.769	2201.535	40,562	1446	0.465
Liver cancer	2267.930	752.100	13,711	795	0.332
Lung cancer	3407.739	1040.659	17,009	1324	0.305
Non-Hodgkin lymphoma (NHL)	618.754	493.633	7163	174	0.798
Oesophageal cancer	1786.330	507.356	12,515	576	0.284
Ovarian cancer	1111.306	436.080	9173	233	0.392
Pancreatic cancer	2064.473	956.450	21,192	404	0.463
Prostate cancer	1242.767	347.120	6395	320	0.279
Skin cancer	1259.639	542.615	18,179	316	0.431
Stomach cancer	2650.101	989.186	11,038	1077	0.373
Testicular cancer	864.485	258.948	4745	331	0.300
Thyroid cancer	2358.799	1223.593	9165	308	0.519

Notes. The column 2, 3, 4, and 5 list the means, standard deviation, maximum, minimum and CV for the whole decade.

**Table 2:** Descriptive information of 20 cancers’ Baidu Media Index.

Cancer	Means	Standard Deviations	Maximum	Minimum	CV
Bladder cancer	0.878	22.376	1347	0	25.485
Brain cancer	0.617	6.217	309	0	10.076
Breast cancer	13.729	43.669	1775	0	3.181
Cervical cancer	5.592	26.033	1090	0	4.655
Colorectal cancer	0.137	0.588	9	0	4.292
Endometrial cancer	0.174	0.736	20	0	4.230
Head and neck cancer	0.001	0.023	1	0	23.000
Kidney cancer	0.537	1.833	51	0	3.413
Leukemia	48.771	84.349	1547	0	1.729
Liver cancer	8.034	22.135	754	0	2.755
Lung cancer	16.500	114.535	6563	0	6.942
Non-Hodgkin lymphoma (NHL)	0.022	0.190	5	0	8.636
Oesophageal cancer	1.180	4.512	220	0	3.824
Ovarian cancer	1.036	3.253	96	0	3.140
Pancreatic cancer	1.775	4.565	148	0	2.572
Prostate cancer	2.561	14.008	644	0	5.470
Skin cancer	1.090	7.029	304	0	6.449
Stomach cancer	7.616	27.309	913	0	3.586
Testicular cancer	0.153	0.740	21	0	4.837
Thyroid cancer	1.406	5.206	144	0	3.703

Notes. The column 2, 3, 4, and 5 list the means, standard deviation, Max & Min and CV for the last decade.

**Table 3:** The Granger causality results (2011–2020).

	2011		2012		2013		2014		2015		2016		2017		2018		2019		2020	
	Media → Public F-Statistic	Public → Media F-Statistic	Media → Public F-Statistic	Public → Media F-Statistic	Media → Public F-Statistic	Public → Media F-Statistic	Media → Public F-Statistic	Public → Media F-Statistic	Media → Public F-Statistic	Public → Media F-Statistic	Media → Public F-Statistic	Public → Media F-Statistic	Media → Public F-Statistic	Public → Media F-Statistic	Media → Public F-Statistic	Public → Media F-Statistic	Media → Public F-Statistic	Public → Media F-Statistic	Media → Public F-Statistic	Public → Media F-Statistic
Bladder	1.180	1.023	0.698	0.537	2.157 *	1.864	85.225 ***	1.981	72191.3 ***	0.144	0.724	0.486	/	/	/	/	/	/	1.047	1.958
Brain	9.927 **	0.003	3.789 **	1.373	1.247	3.452 **	64.460 ***	1.867	6.549 **	0.395	4.675 ***	0.477	3.398	0.405	2.200	5.323 *	/	/	/	/
Breast	5.773 ***	3.213 **	11.624 ***	2.562 *	2.781 **	0.359	8.141 ***	0.787	375.451 ***	8.141 ***	0.871	0.257	6.584 ***	3.395 **	14.424 ***	2.313 *	0.753	0.987	2.693 **	2.015 *
Cervix	4.381 ***	2.398 *	1.974 *	2.039 *	2.342 *	8.901 ***	1.976	0.766	3.228 **	2.128 *	44.201 ***	16.417 ***	34.097 ***	1.487	0.633	1.024	1.026	1.719	0.773	3.918 **
Colorectal	/	/	/	/	0.167	0.275	31.401 ***	0.329	0.091	0.265	/	/	/	/	/	/	/	/	/	/
Endometrium	1.899	2.330 *	1.815	2.605 **	1.340	1.237	2.020*	0.330	0.235	1.022	4.800 **	5.976 ***	/	/	/	/	/	/	/	/
Head & neck	/	/	/	/	/	/	/	/	/	/	/	/	/	/	/	/	/	/	0.115	55.795 ***
Kidney	1.822	1.697	1.509	0.947	3.158 **	1.308	11.775 ***	1.026	2.422 *	1.279	/	/	/	/	2.522 *	1.070	/	/	/	/
Leukemia	6.378 ***	3.334 **	7.645 ***	1.687	26.041 ***	1.907	32.081 ***	4.850 ***	57.203 ***	3.135 **	6.174 ***	0.912	4.551 **	1.226	5.292 **	4.238 *	4.255 ***	3.768 ***	0.583	2.335
Liver	5.019 ***	2.058 *	8.869 ***	2.585	7.160 ***	1.685	2.955 **	1.736	147.958 ***	0.652	1.498	1.013	1.390	0.310	55.942 ***	3.374 ***	1.664	2.549 *	3.829 ***	1.475
Lung	5.740 ***	2.268 *	7.328 ***	1.328	45.869 ***	4.542 *	18.820 ***	2.673 *	0.041	0.009	1.879	1.450	2.896 **	4.724 ***	1.619	1.896	76.983 ***	6.283 **	/	/
Lymph	0.310	5.228 ***	0.276	0.381	3.369	0.024	0.492	1.163	0.765	11.528 ***	1.129	1.303	/	/	/	/	/	/	/	/
Oesophagus	2.169 *	2.601 **	2.092 *	0.651	7.572 ***	2.211 *	1361.56 ***	0.051	2.962 **	1.116	/	/	1.775	1.116	1.739	4.129 ***	0.637	1.704	/	/
Ovary	2.244 *	1.219	1.156	1.739	5.446 ***	1.301	2.067	0.948	5.998 ***	4.200 ***	2.685	0.077	/	/	3.919 ***	5.175 ***	/	/	0.380	2.046
Pancreas	45.599 ***	26.014 ***	3.769 ***	0.675	1.276	1.343	2.141 *	2.208 *	3.913 ***	2.109	18.179 ***	0.550	2.037 *	1.716	0.130	1.423	4.623 ***	1.108	1.387	1.424
Prostate	1.353	2.413 *	294.085 ***	1.193	4.876 ***	1.518	1.168	5.028 ***	4.212 ***	2.859 **	4.098 ***	1.841	1.435	3.294 **	0.864	1.587	1.281	2.982 ***	/	/
Skin	1.279	2.189 *	425.442 ***	0.154	5.353 ***	1.017	8.327 ***	0.667	2.047	2.795 *	3.463 *	1.157	0.671	2.353 *	/	/	/	/	/	/
Stomach	11.477 ***	1.7522	26.739 ***	1.507	2.201 *	5.586 ***	8.583 ***	0.801	13.895 ***	3.023 *	196.907 ***	0.143	2.679 **	2.065 *	4.319 ***	2.072 *	4.328 ***	3.237 **	1.011	2.209 *
Testis	1.776	0.926	3.007 *	3.367 *	6.337 ***	1.165	39.096 ***	0.834	0.611	0.102	/	/	/	/	/	/	/	/	/	/
Thyroid	2.886 **	0.490	2.134*	0.442	2.504 *	1.684	11.369 ***	1.526	3.531 ***	1.746	4.845 ***	2.008 *	0.927	1.647	0.502	3.060**	2.025*	2.030*	1.862	1.133

Note. According to the ADF test, some series have a unit root, and after the first-order difference, all series have become stationary, meeting the requirements for the Granger causality test. * *p* < 0.05. ** *p* < 0.01. *** *p* < 0.001.

**Table 4:** Details about six fluctuations of the Baidu Search Index.

Cancer/Year	Main Causes	Peak of Baidu Search Index/Date	Peak of Baidu Media Index/Date
Pancreatic Cancer/2011	Death of foreign celebrity	21,192/6 October 2011	148/10 October 2011
Skin Cancer/2012	Diagnosis of domestic celebrity	18,179/25 October 2012	304/23 October 2012
Breast Cancer/2015	Death of domestic celebrity	313,389/16 January 2015	1775/15 January 2015
Bladder Cancer/2015	Death of domestic celebrity	44,617/16 March 2015	1347/15 March 2015
NHL/2015	Cancer-related film	5709/20 August 2015	5/20 August 2015
Head and Neck Cancer/2020	Scientific advance	3355/11 February 2020	1/13&15 February 2020

Note. The column 2 lists the main causes of the Baidu Search Index fluctuations, marked by Baidu for all annual peaks of the Baidu Search Index.

## Data Availability

Data sharing is applicable to this article on request from the corresponding author.
